# High Rate of Postural Blindness in Patients With Idiopathic Parkinson's Disease: A Clinical Observation

**DOI:** 10.1155/padi/9272217

**Published:** 2025-07-20

**Authors:** Damiano D. Zemp, Daria Dinacci, Salvatore Galati

**Affiliations:** ^1^Geriatric Service, Ospedale Regionale di Mendrisio, EOC, Mendrisio, Switzerland; ^2^Neurocenter of Southern Switzerland, EOC, Lugano, Switzerland; ^3^Clinica Hildebrand Centro Riabilitazione Brissago, Brissago, Switzerland; ^4^Faculty of Biomedical Sciences, Università della Svizzera Italiana (USI), Lugano, Switzerland; ^5^Movement Disorders Unit, Neurocenter of Southern Switzerland, EOC, Lugano, Switzerland

**Keywords:** Parkinson, postural sway, Romberg Quotient

## Abstract

**Background:** Patients affected by idiopathic Parkinson's disease (IPD) are known to have difficulties in sensorial integration. The ratio of the postural sway in the standing position with closed eyes to open eyes (Romberg Quotient) is a simple way to investigate the role of the visual channel in postural control in this category of patients.

**Objective:** We aim to share our observation about the incidence in patients with IPD of postural blindness, namely the reduction of the postural sway by closing the eyes.

**Methods:** Patients had to stay quiet on a force plate for 30 s in four conditions: eyes open and closed both on a firm and a compliant surface.

**Results:** 30% of the 22 patients analyzed reduced their postural sway by closing their eyes on both firm and compliant surfaces.

**Conclusion:** The role of vision for postural control in patients with IPD should be further investigated.

## 1. Introduction

In late 2018, the movement disorders clinic at the Neurocenter of Southern Switzerland (NSI) Ente Ospedaliero Cantonale (EOC) in Lugano, Switzerland, implemented a computerized postural analysis for idiopathic Parkinson's disease (IPD) patients to evaluate whether such an instrumental analysis could contribute to a better patient intake by clinicians and rehabilitation therapists.

In clinical practice, force plates are used to analyze postural control and the interaction between the visual, proprioceptive, and vestibular systems [1]. During this type of analysis, patients are required to stand for a defined period (e.g., 30 s) under different conditions (e.g., eyes open [EO]/eyes close [EC], firm surface [FS]/compliant surface [CS]), whereas the movement of the center of pressure (COP) is measured [2].

As patients with IPD experience not only movement control disorders but also problems with sensorial integration [3, 4], computerized postural analysis seems to be a valuable tool for evaluating their postural control strategy [5].

The Romberg Quotient (RQ) measures how much the eyes contribute to maintaining balance. It is calculated by dividing the closed-eyes value by the open-eyes value. In healthy subjects, we generally observe RQs = 2, meaning that the eyes have an active role in the postural sway control [6, 7]. The higher the RQ, the higher the dependence on vision. Low RQs (1-2) are found in patients in whom the visual channel does not have a primary role in postural control. For RQs ≤ 1 Marucchi and Gagey introduced in 1987 the definition “postural blindness” (PB) [8]. In patients with PB, the visual channel does not support the postural control or even acts as a disturbance factor.

## 2. Materials and Methods

For the postural static posturography test, we used a piezoelectric force plate with a standardized protocol for geriatric patients. The protocol provides four conditions of a duration of 30 s each, combining EO and EC on a FS and a CS [9]. Participants were instructed to remain still and silent. A visual target was positioned at eye level, 2 m away. No biofeedback was available. The time from the intake of the last levodopa doses to the start of the assessment was not standardized. In the patients analyzed, it was between 0 and 4.5 h (mean 2.5 h).

## 3. Results

The general characteristics and the stability parameters of the 22 patients of our cohort are described in Table 1. Compared to the data of our geriatric patients published elsewhere [9, 10] and to healthy elderly [11], the mean sway area (SA) and the mean sway velocity resulted to be higher and coherent with previous findings [12–15], but discordant with Mirahmadi et al. [16], who found no difference between the stability of patients with Parkinson's disease and healthy control subjects based on excursion of COP, and discordant with Raethjen et al. [17], who found lower sway parameters in Parkinson populations and a better posturographic performance with explicit visual feedback of COP position.

**Figure 1:**
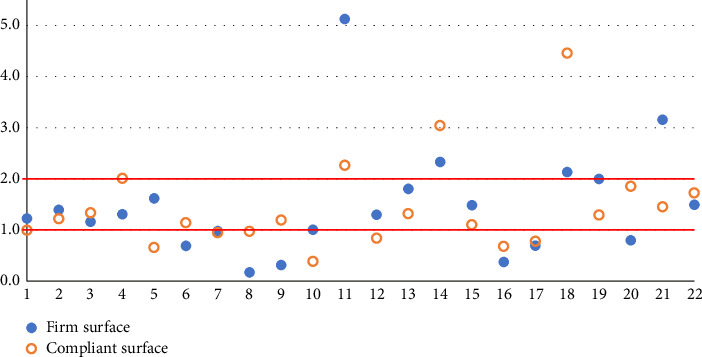
Romberg Quotient for sway area of the 22 patients. The *X*-axis lists the 22 patients. The *Y*-axis represents the Romberg Quotient of the sway area.

When analyzing our cohort's SA RQ, we observed that six patients had PB on FS and eight on CS. Only three patients on FS and four on CS had a RQ > 2 (Figure 1). Some other authors found a mean RQ < 1 [14, 15] or very low RQs (1.1-1.2) [12, 16], indicative of PB in IPD. The sway parameters were heterogeneous, and PB was observed in participants with both well-controlled postures and those exhibiting normal or even high levels of instability, measured by both the FS and the CS. This suggests that sway (in)stability does not serve as a predictor for PB. In addition, we did not find any correlation between the total score on the MDS-UPDRS Part III and PB.

We found two other studies with higher RQs that are similar to healthy elderly [13, 17]. In a previous study [18], the subjective visual vertical test (rod and frame test) and static posturography were studied in IPD. In this study, the RQ did not differ between IPD and controls, even if IPD resulted in visual dependence in the rod and frame test, but the RQ value and the IPD errors in estimating subjective visual vertical had a direct correlation.

Therefore, there is no evidence that patients with IPD with a Hoehn and Yahr (H&Y) score ≤ 3 are generally more dependent on the visual channel for maintaining static balance. On the contrary, it seems that PB is quite common in IPD.

However, all the studies mentioned reported the mean RQ. We found no study that stratified the RQ value. So, it is unclear how common PB is in different populations.

## 4. Discussion

These data contrast the general idea that IPD patients have a poor kinesthetic integration ability associated with visual dependence, as suggested by different subjective postural vertical analyses before. In detail, IPD patients have been shown to recognize worse than control upper limb and trunk positions and movements [19–22]; on the other hand, IPD patients were demonstrated to be visual dependent in subjective postural vertical and subjective visual vertical judgment [23–25].

Even if we have this “visual dependence” evidence in specific settings in IPD, we also know that visual and visual–spatial symptoms are common in IPD [26–28] and are known to affect postural stability, gait control, and the perception of space [29]. The most common visual disorders are retinal dysfunction caused by reduced dopamine release that leads to several visual disturbances such as shorter duration of negative afterimages, disruption of processing at the level of the retina, oculomotor changes, reduced depth perception, inability to inhibit attention toward peripheral objects not relevant for the task, and more difficulties for detecting the direction of motion [30]. In addition to vision, the processing of the visual information acquired plays a central role in postural control, and it is impaired in IPD, especially in those patients affected by cognitive deficits [31]. Moreover, a recent study observed that patients with IPD overestimate the reliability of visual information [32].

The instruction we gave (stay still) could be interpreted as taking a comfortable position that could lead to more micromovements with OA and a more rigid posture for reducing sway as a safety-first strategy with OC that needs a higher muscle tonus but is less comfortable.

The interpretation of these seemingly conflicting data is complex. One explanation could rely on a possible IPD deficit in the automatic sensory input relevance and ponderation analysis for a correct shift required for postural control. In this regard, levodopa intake could reinforce this phenomenon in the first hours after intake [22].

Previous studies showed that IPD has an impaired ability to reweight various sensory inputs controlling posture and shifting from a visual to a proprioceptive control of postural sway with contextual hyperactivity of the visuopostural loop [33]. In this context, explicit visual feedback of COP, as in Raethjen et al. [17], could help patients better focus on multisensory integration, allowing for improved posturographic performance. Furthermore, eight of our patients showed PB on CS, which means that IPD is disturbed by visual input not only when IPD is using predominantly somatosensory information but also when using more vestibular information, suggesting a general impairment in sensory gating in IPD and good use of vestibular function again [34].

The combination of visual impairment, difficulties in sensorial information processing, and high confidence in the eyesight can possibly explain PB, as vision can act as a disturbance factor during static stance. Closing the eyes eliminates ocular interference, improving postural control.

## 5. Conclusion

In conclusion, we believe that further investigation should be conducted to (1) examine the prevalence of PB in different H&Y stages of IPD and compare it with other populations, (2) explore any relation between PB and the aforementioned visual problems, and (3) determine if PB can be treated and if patients with IPD can benefit in terms of security from such a treatment.

## Figures and Tables

**Table 1:** Sample description.

	Patients (*n* = 22)
General characteristics	
Age (years)	70.2 ± 9.5 [54–86]
Female/male (*n*)	8/14
Hoehn and Yahr Scale (points)	2.1 ± 0.4 [1.5–3.0]
Stability parameters	
Firm surface	
Sway area (cm^2^)	4.2 ± 3.6 [0.4–14.6]
Sway velocity (cm/s)	4.0 ± 1.8 [1.2–8.0]
Romberg Quotient	1.5 ± 1.1 [0.2–5.1]
Compliant surface	
Sway area (cm^2^)	7.4 ± 5.0 [2.1–22.3]
Sway velocity (cm/s)	4.3 ± 2.0 [1.5–10.6]
Romberg Quotient	1.4 ± 0.9 [0.4–4.5]

## Data Availability

The data that support the findings of this study are available from the corresponding author upon reasonable request.
